# Hypothesis on etiopathogenesis, congenital or acquired, of an imperforate distal ureter: a case report

**DOI:** 10.1186/s13256-015-0711-8

**Published:** 2015-10-07

**Authors:** Vincenzo Bagnara, S. Castorina, S. Gerocarni Nappo, G. Privitera, T. Luca, P. Caione

**Affiliations:** 1 Department of Maternal and Infant Medicine and Radiological Sciences, University of Catania, Via S. Sofia, 78, 95123 Catania, Italy; 2 “G.B. Morgagni” Mediterranean Foundation, 95125 Catania, Italy; 3 Department of Biomedical and Biotechnological Sciences, University of Catania, 95125 Catania, Italy; 4 Division of Urology-Andrology, Department of Paediatric Nephrology-Urology, “Bambino Gesu” Children’s Hospital, Research Institute, Rome, Italy

**Keywords:** Imperforate distal ureter, Pyeloplasty, Ureteral atresia, Ureteropelvic junction obstruction

## Abstract

**Introduction:**

Ureteral atresia is a rare disease usually associated with a non-functioning kidney. Its association with other urinary anomalies is rare.

**Case presentation:**

In this study we discuss the possibility of congenital or acquired etiology of a right imperforate distal ureter. Here we report the case of 11-month-old white boy with a right ureteropelvic junction obstruction. He underwent a right pyeloplasty when he was 11-months old, and 3 weeks after surgery a cystoscopy was performed. Two months after the first operation, he underwent a right ureteral meatoplasty and a new pyeloplasty.

**Conclusions:**

To the best of our knowledge, few cases of imperforate distal ureter have been described in the literature. The suspicion of a non-patent terminal ureter, occurring during upper urinary tract surgery, must be intraoperatively clarified to preserve the renal function and to avoid more complex surgical approaches.

## Introduction

Ureteral atresia is a rare congenital abnormality usually associated with a dysplastic non-functioning kidney [1]. It is hypothesized that it could be caused by a failure of canalization of a segment of ureter during the process of development and elongation of the ureteric bud. The production of urine starts at 9 weeks of development when the ureter joins the urogenital sinus which is still obstructed by the Chwalla’s membrane. This membrane decreases during approximately the 37th to 47th days of pregnancy [2, 3]. Ureteral atresia is often a cause of hydronephrosis, a distension and dilation of the renal pelvis and calyces caused by the obstruction of urine from the kidney. Prenatal diagnosis of hydronephrosis is possible and, in fact, most cases in pediatric patients are incidentally detected by routine screening ultrasounds obtained during pregnancy [4]. The incidence of prenatal hydronephrosis is 0.59 to 1.4% [5]. The association of distal ureteral atresia with ureteropelvic junction (UPJ) obstruction has never been described in the literature; the former is normally correlated with renal dysplasia.

## Case presentation

**Fig. 1:**
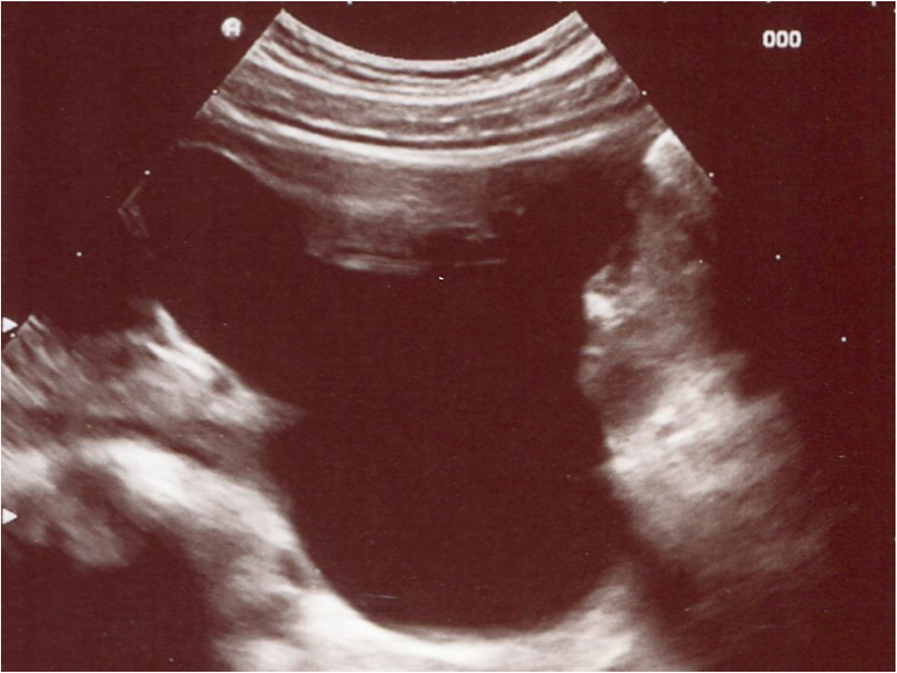
Longitudinal ultrasound image of right renal pelvis

**Fig. 2:**
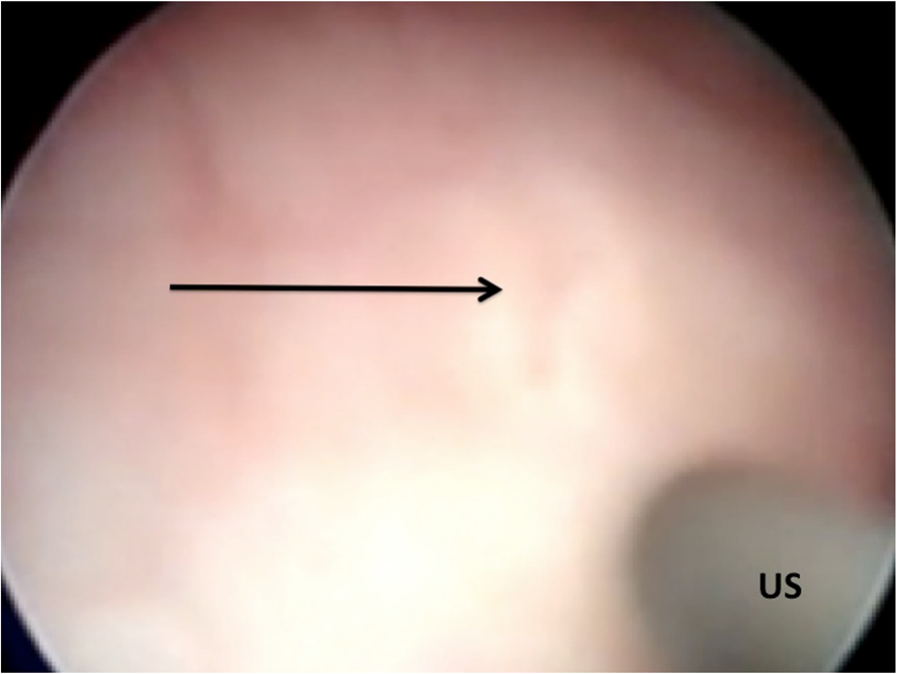
Attempt of catheterization of presumed meatus (*arrow*) with ureteral stent (*US*)

**Fig. 3:**
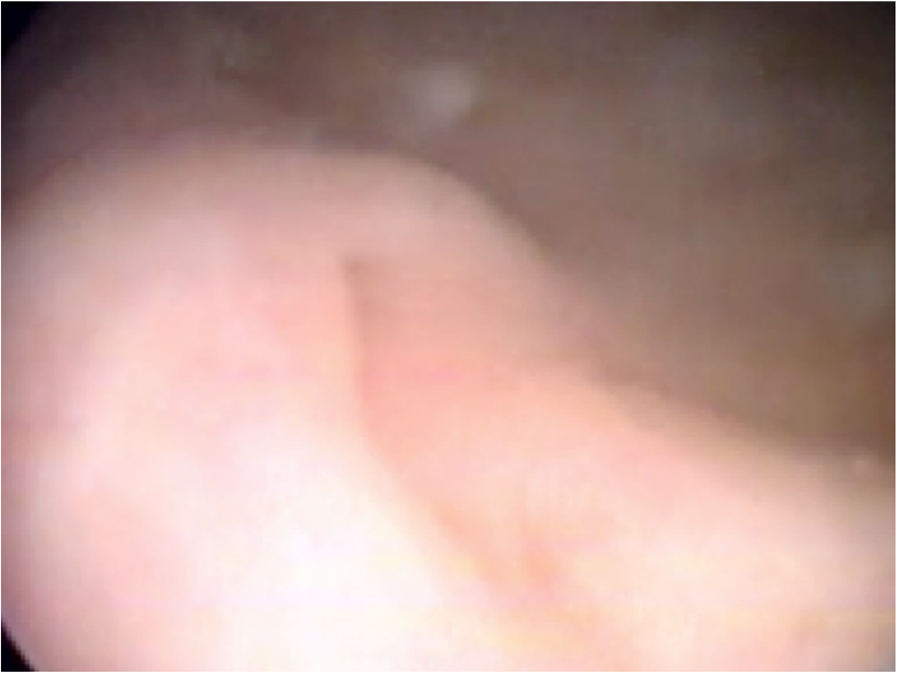
Appearance of the right ureteral meatus after JJ stent removal

**Fig. 4:**
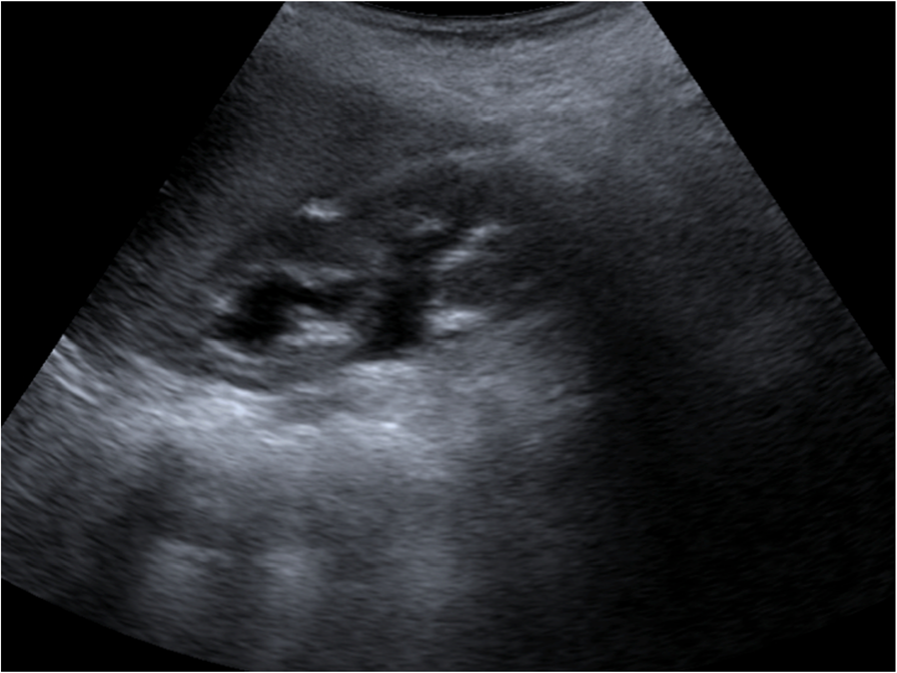
Ultrasound image 30 months after surgery

A newborn baby boy was referred to our department with a prenatal diagnosis of bilateral hydronephrosis. The evaluation included two ultrasonographic studies when he was 2- and 6-months old and a sequential functional scintigraphy with technetium-99m-diethylene-triamine-pentaacetic acid (DTPA) study at the age of 3 months. Sequential functional scintigraphy showed a right UPJ obstruction with a split function of 37% for his right kidney. Interpretation of imaging studies was made from the original radiologists’ reports and from the original images (Fig. 1). When he was 11-months old, he underwent Anderson-Hynes right pyeloureteroplasty. As the JJ ureteral stent was impossible to insert through the ureterovesical junction, a pyelostomy was performed with a 8 Ch Foley catheter. Postoperative radiographic transnephrostomic controls did not permit a view of his right ureter, probably due to anastomosis edema. For this reason, 3 weeks after surgery, a JJ stent was placed by cystoscopy, but it was impossible to visualize the right ureteral meatus (Fig. 2). Subsequently, micturition cystography showed only a left vesicoureteral reflux (VUR). Two months later, the patient underwent a right posterolateral lombotomy on the existing scar. The UPJ was found and the ureter under it was dissected, but it was impossible to pass ureteral probes from his ureter to his bladder. For this reason, a median cystostomy was performed and a Pollack probe was introduced in the ureter by the proximal ureterotomy, to carry out the right ureteral meatoplasty with a 4,7 Ch JJ ureteral stent. The pyeloureteral anastomosis completed the surgical operation. Ten weeks after the second surgery, the baby underwent cystoscopy to remove the JJ stent (Fig. 3). During the same procedure, VUR correction was performed by subureteral injection of dextranomer/hyaluronic acid copolymer as bulking agent. During the follow-up, a healthy renal function and a normal excretory function of both kidneys were confirmed by ultrasound scans (Fig. 4) and mercaptoacetyltriglycine (MAG3) renal sequential scintigraphy which showed a split function of 40% for his right kidney. Thirty months after surgery, he is well, he grows up normally and he has had no urinary infections.

## Discussion

A congenital ureteral obstruction can be caused by UPJ obstruction, primitive megaureter, ectopic ureter, ureterocele, intermediate ureter stenosis, ureteral valves and distal ureteral atresia [6, 7]. The latter is one of the rarest congenital anomalies caused by abnormal embryogenesis. Changes in the number or position of buds, occurring during the embryological development, are the causes of anomalies. Therefore, the renal development and the dysplasia are correlated to the position of the ureteral meatus in the vesical trigone [8].

Ureteral atresia derives from a lack of canalization of the ureter caused by an ischemic injury occurring during the elongation of the ureteral bud [9]. This lack of canalization can be unilateral or bilateral and may occur in all the ureter or in some parts of it [10]. In the distal atresia, the ureter terminates at the back of the bladder as a “cul-de-sac” but does not communicate with the bladder itself as occurs in the primitive megaureter, in which an obstructive ureterovesical communication is present and the stenosis is functional. This condition is associated with a kidney dysplasia [11], even if cases of renal function recovery, after removal of the obstruction, are described in the literature [11, 12].

A preoperative diagnosis of ureteral atresia is difficult and in infancy it presents as an abdominal mass caused by a significant dilatation of the ureter proximally to the atresic segment [11, 13]. Fever and leukocytosis may be present [11, 14]. Finally, the atresia of the ureter with no signs of infection in the dysplastic kidney may be unknown up to adulthood or throughout one’s life [1, 9, 14].

In this case we suspected a ureteral atresia, but the lack of ureteral dilatation, associated with a normal function of the kidney, did not allow a preoperative diagnosis of congenital atresia of the ureter. Moreover, its discovery, which occurred after we tried (although delicate and without forcing) to do a catheterization of the ureter for the position of the JJ stent, and the inflammation of the bladder, did not exclude the hypothesis of an acquired obliteration of the ureteral meatus rather than a congenital one. In fact, in babies under 1-year old the ureteral meatus is often small and very sensitive to modest traumas. We also evaluated the hypothesis of the persistence of the Chwalla’s membrane, which can be responsible for an adult ureterocele. In this condition, an anatomically demonstrable communication is not present but a correct renal function can be preserved after many years [8].

In our patient, we did not consider an atresia of ureter but an acquired imperforate ureterovesical junction secondary to a trauma or an inflammation, or due to a congenital persistence of the Chwalla’s membrane. The absence of a segment of the ureter should always be demonstrable to diagnose an atresia. In our opinion, the aspect of the distal ureter which terminates as cul-de-sac is not sufficient to assume the presence of an atresia of the ureter rather than an imperforation. Therefore, distal atresia with a preserved kidney function cannot be considered real if it does not determine a complete obstruction. It is probable that a mechanical obstacle for the catheterization may be present but a functional stop might not be present, as occurs, for example, in the case of persistence of the Chwalla’s membrane, which cannot determine alone a reduction in kidney function. The reason for the maintenance of a normal renal function after birth is difficult to explain and to understand in the presence of a real atresia of the ureter. In fact, most of the cases of ureteral atresia reported in the literature and discovered in neonatal [11] and in adulthood life [1, 9, 11] were treated with nephrourectomy for the presence of a serious renal dysplasia.

## Conclusions

The causes of distal ureteral obstruction are various and they must be considered in the classification and in the treatment of this uncommon pathology. The suspicion of a non-patent terminal ureter, occurring during urinary tract surgery, as verified in UPJ obstruction, must be immediately and intraoperatively clarified to preserve a possible residual renal function and to avoid new and more complex surgical approaches.

## Consent

Written informed consent was obtained from the parents of the patient for publication of this case report and any accompanying images. A copy of the written consent is available for review by the Editor-in-Chief of this journal.

## Abbreviations

UPJ: Ureteropelvic junction
VUR: Vesicoureteral reflux

## References

[1] Alcaraz A, Vinaixa F, Tejedo-Mateu A, Forés MM, Gotzens V, Mestres CA, et al. Obstruction and recanalization of the ureter during embryonic development. J Urol. 1991;145:410–6. 10.1016/s0022-5347(17)38354-4 1988740

[2] Coplen DE. Ureteral obstruction and malformations. In: Ashcraft KW, Holcomb III GW, Murphy JP, editors. Pediatric surgery. 4th ed. Philadelphia: Elsevier; 2005. p. 732–79.

[3] Bhattacharjee PK, Ghosal S, Sharma GD. Distal ureteric atresia presenting as an abdominal lump in an adult. Indian J Surg. 2009;66:175–7.

[4] Estrada CR. Prenatal hydronephrosis: early evaluation. Curr Opin Urol. 2008;18:401–13. 10.1097/MOU.0b013e328302edfe 18520762

[5] Kannaiyan L, Karl S, Mathai J, Chacko J, Sen S. Congenital ureteric stenosis: a study of 17 children. Pediatr Surg Int. 2009;25:513–7. 10.1007/s00383-009-2368-0 19437024

[6] Karanastasis D, Antoniou N, Tsagatakis E, Sakalis K, Stenos I. Distal ureteral atresia associated with ipsilateral renal dysplasia. Scand J Urol Nephrol. 1992;26:77–9. 10.3109/00365599209180401 1631512

[7] Liang CC, Cheng PJ, Lin CJ, Chen HW, Chao AS, Chang SD. Outcome of prenatally diagnosed fetal hydronephrosis. J Reprod Med. 2002;47:27–32. 11838306

[8] Ashimine S, Miyazato M, Hayashi E, Morozumi M, Sugaya K, Ogawa Y. Distal ureteral atresia: recovery of renal function after relief of obstruction at ten months old. Int J Urol. 2005;12:578–80. 10.1111/j.1442-2042.2005.01103.x 15985082

[9] Loyd Jr MS, Scally J, Irwin PP. Incidental detection of a unilateral dilated blind-ending ureter, renal agenesis, and a dilated seminal vesicle. Urol J. 2012;9:639. 23235965

[10] Morozumi M, Ogawa Y, Fujime M. Distal ureteral atresia associated with crossed renal ectopia with fusion. Recovery of renal function after release of a 10-year ureteral obstruction. Int J Urol. 1997;9:512–5. 10.1111/j.1442-2042.1997.tb00295.x 9354956

[11] Riccabona M. Obstructive diseases of the urinary tract in children: lessons from the last 15 years. Pediatr Radiol. 2010;40:947–55. 10.1007/s00247-010-1590-1 20432013

[12] Ruano-Gil D, Coca-Payeras A, Tejedo-Mateu A. Obstruction and normal recanalization of the ureter in the human embryo. Its relation to congenital ureteric obstruction. Eur Urol. 1975;1:287–93. 1233198

[13] Schoenwolf GC, Bleyl SB, Brauer PR. Larsen’s human embryology. 4th ed. Edinburgh: Churchill Livingstone; 2009.

[14] Sinha RS, Bhattacharjee P, Majhi T. Distal ureteric atresia a case report. J Indian Assoc Pediatr Surg. 2002;7:156–8.

